# The burden of diarrhoeal diseases in the Democratic Republic of Congo: a time-series analysis of the global burden of disease study estimates (1990–2019)

**DOI:** 10.1186/s12889-022-13385-5

**Published:** 2022-05-25

**Authors:** Gloire O. Mbaka, Rute Vieira

**Affiliations:** 1 UCLA DRC Health Research and Training Program and Institute of Applied Health Sciences, University of Aberdeen, Aberdeen, Scotland; 2 Medical Statistics Team, Institute of Applied Health Sciences, University of Aberdeen, , Polwarth Building (Room 1.028), Foresterhill Aberdeen, AB25 2ZD Aberdeen, Scotland

**Keywords:** Diarrhoeal diseases, YLDs, Mortality, Prevalence, WASH, Child malnutrition

## Abstract

**Background:**

Diarrhoeal diseases are important causes of disability and mortality being one of the main causes of mortality in the Democratic Republic of Congo (DRC). One of the largest and wealthiest African countries, DRC has been for long subjected to continuous political and economic instability, conflicts and disease outbreaks. This study aimed to address the knowledge gap in understanding how prevalence, mortality and burden of diseases in DRC changed over time and examine the influence of specific factors in these disease-related outcomes.

**Methods:**

A time-series analysis of the Global Burden of Diseases, Injuries, and Risk Factors Study 2019 estimates was performed to describe prevalence, years lived with disabilities (YLDs) and mortality due to diarrhoeal diseases, by age-group and sex, between 1990–2019 in DRC. The contribution of water, sanitation and hygiene (WASH) and child malnutrition risk factors to these outcomes was also analysed. Piecewise regression analysis was used to assess trends over time.

**Results:**

The overall age-standardised prevalence of diarrhoeal diseases for both sexes in DRC was 1350.84 (UI:1240.16—1461.62) cases per 100,000 people in 1990. The prevalence increased until 2019, also fuelled by the movement of Rwandan refugees to DRC and First/Second Congo wars between 1996–2003. Age-standardised prevalence and mortality were consistently higher in males, compared to females, decreasing by 42% and 54%, respectively, between 1990 and 2019. Overall prevalence was also usually higher in over 70 years old, except between 1998–2003 when mortality in under five years old was the highest. Unsafe water sources and child wasting among under five years old were the main contributors to YLDs and deaths associated to diarrhoeal diseases in DRC.

**Conclusion:**

Diarrhoeal diseases are important and preventable causes of disability and mortality in DRC. National measures of surveillance and cost-effective interventions targeting the identified risk groups could be effective in reducing its prevalence and associated burden.

**Supplementary Information:**

The online version contains supplementary material available at 10.1186/s12889-022-13385-5.

## Background

Diarrhoeal diseases are major causes of death in developing countries and the third leading cause of death among children, after pneumonia and preterm birth complications [1]. Globally, there were 1.6 million deaths from diarrhoeal diseases in 2019 (Supplementary Fig. 1), with children less than five years old being the most affected with around 25% and 31% of deaths in Africa and South Asia respectively [1, 2]. Diarrhoeal diseases are caused by a host of bacterial, viral and parasitic organisms spread by water contaminated with faeces mostly. Rotavirus and *Escherichia coli*, remain the most common etiological agents of moderate-to-severe diarrhoea in developing countries, with pathogens like *cryptosporidium* and *shigella* species also having an important role [3, 4].

Diarrhoeal diseases are one of the main public health concerns in the Democratic Republic of Congo (DRC) with over 1.45 millions of people having the condition in 2019, and an extremely high death rate for under five years old. As of 2013, 119 deaths per 1,000 live births were reported, surpassed only by seven countries in which diarrhoeal diseases accounted for about 11% of child mortality [6]. In 2019, the country reported 6.03% (UI: 3.05—10.05) of overall deaths for all ages for both sexes, due to diarrhoeal diseases [5]. DRC is the largest French speaking country in Africa, rich with natural resources, and has approximately 80 million inhabitants of whom less than 40% live in urban areas [7]. It is still in the process to recover from a series of conflicts and disease outbreaks since the 1990s (Table S1) and deterioration of the macroeconomy [8]. These have also weakened the national health system over the years together with lack of investment and intentional direct attacks from armed groups in conflict areas [9].

According to WHO, the lack of access to health services in DRC, worsened by the deteriorating living conditions, violence and ongoing displacement, will account for a significant rise in the level of morbidity and mortality for all diseases in a near future [10]. In particular, reduced adequate sanitation and hygiene, as well as unsafe water for drinking, cooking and cleaning, are associated with an increase of diarrhoeal diseases prevalence [11]. Globally, 2.2 billion people did not have adequate drinking water services, 4.2 billion people lacked adequate sanitation services, and 3 billion lacked basic handwashing facilities in 2017 [12]. According to UNICEF, 33 million people in rural areas did not have access to quality water in DRC, and only 52% of the population had access to adequate water sources and 29% to adequate sanitation facilities [13]. In 2012, only 16% of total households had access to adequate sanitation, with 4% in rural areas compared to 36% in urban areas. This was considerably less than the average of 44% and 24% in urban and rural areas in sub-Saharan Africa, respectively [6].

Although most diarrhoeal diseases are treatable, several factors affect recovery, in particular childhood malnutrition. In DRC, 43% of children between 0 and 5 years-old are affected by chronic malnutrition [7], with an increase of between 18 and 23% when there is a movement of refugees [14]. In 1991, the WHO and UNICEF launched the Baby-Friendly Hospital Initiative (BFHI) to ensure that all maternities and hospitals become centres of breastfeeding support and improve children nutrition from birth [15]. In DRC, BFHI was an integral part of a national campaign to support breastfeeding in the early 2000s. However, only 25 out of 6000 eligible health facilities in the country, including 13 in Kinshasa, received the certification and, according to the demographic health survey 2013–2014, about 48% of under 6-months children were exclusively breastfed, whilst 79% of children between the age of 6–9 months received complementary foods [16, 17].

Even though epidemics control remains a priority for DRC, the country is still threatened by re-emergence of communicable diseases and there is limited understanding of the contribution of specific risk factors (child malnutrition and water, sanitation and hygiene (WASH)) to mortality and disease burden. Previous studies have investigated the maternal determinants of diarrhoea in children under five years old [18], performed a molecular surveillance of rotavirus infection in the country [19], explored the relationship between the maternal behavioural risk factors to severe diarrhoea [20], and investigated the associations between household latrines and the prevalence of diarrhoea in Idiofa, in DRC [21]. However, to our knowledge, there are no comprehensive studies available investigating trends over time in prevalence, mortality and disability burden of diarrhoeal diseases in DRC and exploring the influence of maternal child malnutrition and WASH risk factors in these health outcomes.

## Methods

### Study design and participants

This study presents an analysis of estimates of specific disease-related outcomes, reported by the Global Burden of Diseases, Injuries, and Risk Factors Study 2019 (GBD 2019), a comprehensive and updated worldwide epidemiological study aiming at the quantification of mortality, morbidity and disability of major diseases, injuries, and risk factors by location, sex, age group, and life years [22]. The metrics and approaches used in the GBD 2019 to investigate diarrhoeal diseases have been reported in detail elsewhere [22–25]. Overall, the GBD 2019 provides a comprehensive annual study for estimates of mortality and morbidity for 369 diseases and injuries as well as Health Life Expectancy (HALE), and 87 risk factors for 204 countries and territories from 1990 to 2019 [24, 25]. The original data used in this study can be downloaded from the GBD website [26].

### GBD statistical methods

We used the estimated results from the GBD Study 2019 for exploring analysing and time trends of prevalence, mortality and burden of diarrhoeal disease in DRC from 1990 to 2019. The GBD 2019 Study estimated these using a spatiotemporal Gaussian process regression available in the Bayesian meta-regression software DisMod-MR version 2.1. Exposure data used in these estimations were extracted from randomized controlled trials, cohorts, pooled cohorts, household surveys, census data, satellite data, disease registries, air pollution monitors, disease notification, civil registration and vital statistics and other sources available to GBD Study 2019 from DRC [24]. Estimates and their 95% uncertainty intervals (UI), generated using the 25th and 975th ordered 1000 draw values of the posterior distribution, were reported. Only estimates for DRC as a whole country were available.

The GBD comparative risk assessment framework was used to obtain the estimates of overall levels in exposure (prevalence), attributable deaths (mortality), and attributable years lived with disability (YLDs), by age group, sex, and year, as well as for WASH and childhood malnutrition risk factors, from 1990 to 2019. Population-wide estimates were calculated as age-standardised health outcomes using the GDB standard population whilst unstandardised rates were used when stratifying by age group. The GBD Study 2019 estimated the portion of diarrhoeal diseases related deaths and YLDs that could be attributed to a given risk factor using the counterfactual scenario of theoretical minimum risk exposure level (TMREL) [25].

### Risk factors

For WASH risk factors, we investigated over time changes in the isolated and combined contribution of unsafe water sources, unsafe sanitation, and no access to handwashing facility to mortality and YLDs by diarrhoeal diseases. For children malnutrition, we investigated over time changes in the effect of suboptimal breastfeeding, non-exclusive breastfeeding, discontinued breastfeeding, child underweight, child wasting and child stunting in the disease-related outcomes, only for the population of children under five years-old.

### Statistical analysis

Based on visual analysis of the time trends, these were not linear and therefore we used piecewise regression analysis to describe and assess the statistical significance of trends over time for the outcomes of interest, as suggested by Boyle and Parkin (1991) [26]. For the purpose of this analysis, time was partitioned into intervals and a separate regression line was fit to each interval. The boundaries between the segments were selected based on the estimates trends behaviour that seemed to be affected by two different processes. For prevalence and YLDs, visual breakpoints existed in 1996 and 2004 (start of First Congo War and end of Second Congo War) and therefore separate regression lines for the periods 1990–1995, 1996–2003, and 2004–2019 were estimated. Mortality, on the other hand, was analysed between 1990–1999 and 2000–2019 as only one breakpoint was observed in 2000. Percentage change was also used to describe the overall change between different years. All analysis were conducted using SPSS v27.

## Results

In 1990, the age-standardised prevalence of diseases for both sexes was approximately 1350.84 (UI: 1240.16 – 1461.62) cases per 100,000 population in DRC. Although there was a 41.01% increase in prevalence rate between 1990 and 2019, this increase was not linear, with significant variation over time as showed in supplementary Fig. 2A and Table 1.

**Table 1:** Piecewise regression coefficients describing trends over time for age-standardised prevalence, YLDs and deaths rate per 100,000 for both sexes, for diarrhoeal diseases in DRC from 1990 to 2019

	**PREVALENCE**			**YLDs**			**DEATHS**	
Timepoints	**1990 – 1995**	**1996 – 2003**	**2004 – 2019**	**1990 – 1995**	**1996—2003**	**2004 – 2019**	**1990—2000**	**2001 -2019**
Coefficients (95% CI)	0.97 (-0.77, 2.70)	40.34 (37.65, 43.04)*)*	10.49 (8.71, 12.27)*	0.13(-0.07, 0.33)	4.51 (4.23, 4.79)*	1.23 (1.04, 1.42)*	2.69 (2.14, 3.25)*	-4.08 (-4.39, -3.77)*
*P*-value	0.195	< 0.001	< 0.001	0.148	< 0.001	< 0.001	< 0.001	< 0.001

^*^Statistically significant: (*). *YLDs* Years Lived with Disability

Between 1990 and 1995, the age-standardised prevalence remained constant, increasing significantly after 1996 by 40 cases per 100,000 per annum until 2003, and by 10 cases per 100,000 per annum thereafter (Table 1). The age-standardised prevalence of diarrhoeal diseases was considerably higher in males, with also a greater increase across the years (46.15%) compared to females (33.96%) (Supplementary Fig. 2A). Among all the age groups (Supplementary Fig. 2B), the prevalence of diarrhoeal diseases was highest in the over 70 years old, reaching a maximum of 5401.96 (UI: 4768.92—5899.70) cases per 100,000 in 2019 with a percentage change (p.c) of + 13.80% between 1990 and 2019. They were followed by the under five years old (p.c + 9.23%), 50–69 (p.c + 64.24%), 15–49 (p.c + 80.18%), and 5–14 (p.c + 58.67%). The trends over the years were mostly linear over time for all age groups, expect in the under five years old, which showed a steep increase between 1996 and 2003 (Supplementary Fig. 2B and Table 2).

**Table 2:** Piecewise regression coefficients describing trends over time for prevalence, YLDs and deaths rate per 100,000 for both sexes by age groups for diarrhoeal diseases in the DRC from 1990–2019

	**PREVALENCE Coefficient (95% CI)**			**YLDs Coefficient (95% CI)**			**DEATHS Coefficient (95% CI)**	
**Age groups**	**1990—1995**	**1996—2003**	**2004—2019**	**1990 – 1995**	**1996—2003**	**2004—2019**	**1990—2000**	**2001—2019**
Under 5	1.19 (-3.28, 5.66)	162.29 (132.21, 192.37)*	-61.76 (-73.30, -50.22)*	0.22 (-0.34, 0.77)	18.53 (15.18, 21.88)*	-6.82 (-8.16,-5.48)*	29.99 (25.04, 34.94)*	-25.39 (-26.76, -24.02)*
***P*-value**	0.501	< 0.001	< 0.001	0.335	< 0.001	< 0.001	< 0.001	< 0.001
5 – 14 years	-6.03 (-9.02,-3.03)*	22.21 (18.93, 25.48)*	8.65 (6.55, 10.74)*	-0.70 (-1.10, -0.30)*	2.55 (2.16, 2.93)*	1.02 (0.78, 1.25)*	0.03 (-0.04, 0.10)	-0.08 (-0.13, -0.04)*
***P*-value**	0.005	< 0.001	< 0.001	0.008	< 0.001	< 0.001	0.372	0.002
15 – 49 years	1.79 (1.09, 2.46)*	20.60 (16.01, 25.19)*	16.71 (14.15, 19.27)*	0.20 (0.13, 0.27)*	2.28 (1.76, 2.80)*	1.92 (1.64, 2.20)*	%1.%2(-0.04, 0.07)	-0.26 (-0.30, -0.22)*
***P*-value**	0.002	< 0.001	< 0.001	0.001	< 0.001	< 0.001	0.629	< 0.001
50 – 69 years	8.48 (6.76, 10.20)*	40.11 (31.10, 49.12)*	34.15 (28.29, 40.00)*	0.94 (0.75, 1.13)*	4.33 (3.37, 5.29)*	3.81 (3.20, 4.42)*	0.08 (-0.11, 0.27)	-2.72 (-2.88, -2.56)*
***P*-value**	< 0.001	< 0.001	< 0.001	< 0.001	< 0.001	< 0.001	0.347	< 0.001
+ 70 years	25.34 (23.58, 27.10)*	45.75 (39.56, 51.94)*	4.65 (2.56, 6.74)*	2.46 (2.24, 2.67)*	4.78 (4.22, 5.35)*	0.62 (0.38, 0.86)*	2.53 (1.36, 3.71)*	-11.58 (-13.27, -9.90)*
***P*-value**	< 0.001	< 0.001	< 0.001	< 0.001	< 0.001	< 0.001	0.001	< 0.001

Statistically significant: (*), *YLDs* Years Lived with Disability

### Mortality and diarrhoeal diseases

In 1990, diarrhoeal diseases were ranked as the 5^th^ major cause of death in DRC with an age standardised mortality rate, for both sexes, of 110.44 (UI: 79.12—160.58) per 100,000 (Supplementary Figs. 3 and 4A). Although there was a 48.04% decrease in mortality between 1990 and 2019, with a mortality rate of 57.38 (UI: 32.54—99.15) per 100,000 in 2019, diarrhoeal diseases were still ranked as the 5^th^ top cause of death in 2019.

Between 1990 and 2000, the number of age standardised deaths caused by diarrhoeal diseases, for both sexes, increased significantly by an average of 3 cases per 100,000 per annum (Table 1). However, Supplementary Fig. 4A shows a specific increase in age-standardised death rate in 1994 which might be related with the refugee movement of Rwandan people, and the related cholera outbreak. This is also noticeable in the supplementary Fig. 4B for children under five years old. Between 2001 and 2019, there was a significant decrease in the overall death rate by an average of 4 cases per 100,000 per annum (Table 1).

The age-standardised death rate was consistently higher for males, decreasing in DRC by 41.47% between 1990 and 2019 and by 54.17% for females. Among all the age groups (Supplementary Fig. 4B), deaths by diarrhoeal diseases were usually higher in the over 70 years old with a percentage change of -36.08%, followed by under five years old (p.c. -60.11%), 50–69 (p.c. -42.77%), 15 – 49 (p.c. -32.45%), and 5 – 14 (p.c. -24.23%). However, mortality in the under five years old increased significantly from the early 1990s until 2000, having the highest mortality of all age groups between 1997–2005, possibly as a result of the 1^st^ and 2^nd^ Congo wars. After 2000, mortality has decreased significantly in all age groups and more significantly in the under five years old (Table 2).

### YLDs and diarrhoeal diseases

In 1990, the age-standardised YLDs rate of diarrhoeal diseases for both sexes was 147.13 (UI: 101.94—200.55) per 100,000 in DRC (Supplementary Fig. 5A), with a 42.47% increase between 1990- 2019 (209.62 (UI: 144.98—291.83) YLDs per 100,000). However, similarly to prevalence and mortality, change over time was not linear. Between 1990 and 1995, there was a statistically significant increase in the number of age standardised YLDs, for both sexes, by an average of 0.13 cases per 100,000 per annum, which then increased equally by an average of 4 cases per 100,000 per annum until 2003. A significant increase in the age standardised YLDs rate of 1.23 cases per annum was found between 2004 and 2019 (Table 1).

The age-standardised YLDs rate for diarrhoeal diseases was higher in males compared to females, with a higher increase across the years observed also in males (47.66%, from 163.65 (UI: 112.65—224.57) per 100,000 in 1990 to 241.65 (UI: 166.19—334.03) per 100,000 in 2019). compared to females (35.23% increase from 132.28 (UI: 91.44—179.76) per 100,000 in 1990 to 178.88 (UI: 123.66—250.24) per 100,000 in 2019). Among all the age groups, the YLDs rate for diarrhoeal diseases was usually higher in over 70 years old (p.c + 14.15%), followed by under five years old (p.c + 9.91%), 50–69 (p.c. + 65.93%), 15–49 (p.c + 81.64%), and 5–14 (p.c + 58.96%), between 1990 to 2019 (Supplementary Fig. 5B). As with prevalence and mortality, children under five years old were the group showing the greatest variation of YLDs rate over time.

### Water, sanitation and hygiene (WASH) risk factors

In 1990, the combined contribution of all WASH risk factors to the age standardised YLDs rate of diarrhoeal diseases was 142.35 (98.27—197.07) YLDs per 100,000 (Supplementary Fig. 6A) with a 41.77% increase on the YLDs rate between 1990 and 2019. Again, there was significant non-linear variation over time (Table 3). WASH factors also contributed considerably to mortality by diarrhoeal diseases (Supplementary Fig. 6B) with unsafe water sources being the main contributor to both YLDs and deaths by diarrhoeal diseases, followed by unsafe sanitation.

**Table 3:** Regression coefficients describing trends over time for age-standardised YLDs and deaths rate per 100,000 for both sexes for diarrhoeal diseases for all WASH risk factors, and child nutrition risk factors for under 5 in the DRC from 1990 to 2019

	**YLDS Coefficient (95%CI)**			**DEATHS Coefficient (95%CI)**	
RISK FACTORS	**1990 – 1995**	**1996 – 2003**	**2004 – 2019**	**1990 – 2000**	**2001 – 2019**
WASH FOR AGE STANDARDISED	0.11 (-0.1, 0.31)	4.41 (4.14, 4.68) *	1.1 (0.92, 1.28)*	2.61 (2.07, 3.16)*	-3.97 (-4.28, -3.67)*
*P*-value	0.221	< 0.001	< 0.001	< 0.001	< 0.001
CHILD MALNUTRITION FOR UNDER-5	0.56 (0.25, 0.86)*	5.0 (4.46, 5.55)*	-3.25 (-3.74, -2.77)*	27.69 (23.15, 32.24)*	-23.43 (-24.61, -22.25)*
*P*-value	0.008	< 0.001	< 0.001	< 0.001	< 0.001

Statistically significant: (*), *YLDs* Years Lived with Disability

Supplementary Fig. 7 shows the contribution of WASH factors to YLDs and mortality rates due to diarrhoeal diseases by sex and age-group in 2019, with WASH factors contributing considerably more to YLDs and deaths in males across all the age-groups as well as over 70 years old and under five years old.

### Child malnutrition risk factors

In 1990, the combined contribution of all child malnutrition risk factors to the YLDs rate for diarrhoeal diseases, among under five year olds, decreased significantly by 2.83%, from 86.11 (UI: 47. 56—134.07) YLDs per 100,000 in 1990 to 83.67 (UI: 43.11—135.03) YLDs per 100,000 in 2019, showing considerable variation over time (Table 3). Supplementary Fig. 8 shows the contribution of each child malnutrition factor to the under five years old YLDs and mortality rates due to diarrhoeal diseases, for both sexes. Across all the child malnutrition factors, non-exclusive breastfeeding was the main contributor to the YLDs rate, and child wasting to the mortality rate by diarrhoeal diseases.

Supplementary Fig. 9 shows the contribution of each child malnutrition factor to the under five years old YLDs and mortality rates by sex in 2019.

### Comparing DRC mortality and YLDs by diarrhoeal diseases with neighbouring countries

The Republic of Angola had the highest prevalence of diarrhoeal diseases in 1990 although it presented a high reduction in age standardised deaths over the years. It decreased from 342.06 deaths per 100,000 in 1990 (UI: 152.15—579.86) to 71.37 deaths per 100,000 (UI: 44.41—116.39) in 2019, with a percentage change of -79.14% (Supplementary Fig. 10B). However, Equatorial Guinea was the country with the greatest reduction in age standardised deaths with a percentage change of -94% from 324.95 deaths per 100,000 (UI: 128.26—593.11) in 1990 to 20.74 deaths per 100,000 (UI: 9.64—40.03) in 2019. The Central African Republic experienced the lowest percentage change of -24%, from 298.42 deaths per 100,000 (UI: 156.81—480.66) in 1990 to 226.15 deaths per 100,000 (UI: 127.31—352.00) in 2019. The Republic of Congo experienced a percentage change of –65%, from 163.71 deaths per 100,000 (UI: 97.63—253.21) in 1990 to 57.53 deaths per 100,000 (UI: 30.48—99.60) in 2019. The Republic of Gabon experienced a percentage change of –71%, from 83.05 deaths per 100,000 (UI: 47.12—132.55) in 1990 to 23.75 deaths per 100,000 (UI: 10.88—44.04) in 2019. Therefore, although the age standardised death rate in DRC was the second lowest after Gabon, its reduction over the years has also been less remarkable. Regarding YLDs, Equatorial Guinea showed the biggest decrease over time although not as steep as the death rate, whilst other countries experienced upward trends (Supplementary Fig. 10A).

## Discussion

This study aimed to describe how prevalence, mortality, and burden of diarrhoeal diseases in DRC changed between 1990–2019 and examine the influence of specific risk factors in these disease-related outcomes.

Although overall prevalence and YLDs increased between 1990 and 2019, mortality has decreased over time. However, these changes were limited and non-linear, with similar change patterns over time found for prevalence and YLDs, relatively constant between 1990 and 1995 and increasing significantly thereafter from 1996 to 2019. These changes in trend seem to relate to events of large-scale population displacement caused by conflicts between ethnic groups in DRC and neighbouring countries. In 1994, there was approximately 850,000 refugees that migrated to Goma, 332,000 to Bukavu, and 62,000 to Uvira. Between 1994–95, these numbers increased with influxes of over 90,000 Burundian refugees after political killings in their country [28]. Pronounced reductions in mortality rate were observed afterwards, possibly due to the impact of surveillance and control program measures for the management of cases, as well as preventive measures and operational training services in the regions affected by outbreaks and wars. Since the beginning of the First Congo war, in 1996, the living conditions of the population in the eastern DRC has drastically worsened [29]. As per our findings, children under five seem to be those mostly affected as mortality increased substantially and surpassed the over 70 s (supplementary Fig. 4B) while all other age groups were mostly unaffected.

Age-standardised prevalence of diarrhoeal diseases and related death rates were higher in males, over 70 years old and under five years old. This finding is consistent with another study that revealed that young children living in developing states in Gaza are at high risk for consequences of diarrhoeal diseases, including dehydration and malnutrition [30]. Access to health care and balanced diets are limited by poverty, [30] and, in DRC, 43% of children are malnourished. To these inequalities add up inequalities in exposure and resistance to disease and therefore children and elderly in poor households are likely to be more exposed to contaminated sources and have poorer health [31].

Unsafe water sources and child wasting were the risk factors that contributed more to the mortality and burden by diarrhoeal diseases in DRC, which is consistent with the highest death rates in over 70 years old and under five years old. Ageing affects physiological fitness (e.g. nutritional status, immune system, renal function) and diarrhoea is caused or is aggravated by comorbidities that are common in the elderly [32]. It was revealed that a longstanding diabetic neuropathy’s complication, such as chronic diarrhoea, might be frequent in older patients as well [32]. These results also showed that the elderly’s death rate by diarrhoeal diseases has not been affected by the interventions in place urging the implementation of targeted interventions especially for elderly in the poorer households. On the other hand, the improvement on death rate observed among the under five years old could be attributed to the national breastfeeding support campaign. This is consistent with a study in Kinshasa that reported that implementing BFHI steps 1–9 statistically decreased diarrhoeal diseases’ prevalence by about 50% [16]. However, DRC has limited means to establish support community groups required for expanding BFHI centres. Nonetheless, previous research demonstrated that BFHI promoted child survival in poor households [16].

Some reduction in overall mortality due to diarrhoeal diseases was observed throughout the years although it is difficult to attribute this decline mainly to the impact of interagency committee measures of control and surveillance or WASH improvement. Issues with data quality can result from DRC’s incomplete medically certified cause of death system.^33^ For effective and sustainable control of diarrhoeal diseases, DRC should develop specific measures of control in line with integrated inter-sectoral public policies already in place. These could be either to improve the social conditions of population in both rural and urban areas, access to adequate water and sanitation, ensured better health care services and health education to all. Access to quality health care should be prioritized for both diagnostics and management of coinfections and other comorbidities that exacerbate the diarrhoeal diseases conditions among elderly. Furthermore, the country should prioritize the investment in research and development of health technologies such as diagnostic tests, drug formulation, vaccines for under five years old, and methods of control for diarrhoeal diseases.

Overall limitations of GDB 2019 study have been published in detail elsewhere [24]. Additionally, the regional effects and cultural beliefs of the population are not captured in this research. As strengths, our study utilized all available data sources in DRC that could be accessed by the GBD Study 2019 in the estimation of trends in the burden of diarrhoeal diseases and associated risk factors. We used results estimated by GBD using standard and robust methodology for estimating the disease-related outcomes. Piecewise regression analysis was used to assess the statistical significance of trends over time, and we used percentage change as well to describe over time change over 29 years in DRC by sex and age.

DRC-specific findings in this research can serve as useful reference to inform policies and programmes for cost-effective interventions to further reduce prevalence and associated burden of diseases in identified risk groups. Additionally, the burden of diarrhoeal diseases findings in DRC can help monitor health progress and evaluate the impact of public health interventions.

## Supplementary Information

**Additional file 1: Supplementary Figure 1. **Illustrating the mortality rate from diarrhoeal diseases in 2019 worldwide [5].

**Additional file 2: Supplementary Figure 2. **Line plots showing age-standardised prevalence of diarrhoeal diseases per 100,000 people, overall and by sex (A) and the overall prevalence by age-group (B), in DRC from 1990 to 2019.

**Additional file 3: Supplementary Figure 3. **Arrow diagram showing the ranking of diseases by burden in DRC from 1990 to 2019 [27].

**Additional file 4: Supplementary Figure 4. **Line plots showing age-standardised death rates for diarrhoeal diseases per 100,000, overall and by sex (A), and by age-groups (B) in DRC from 1990 to 2019.

**Additional file 5: Supplementary Figure 5. **Line plots showing YLDs per 100,000 people, overall and by sex (A) and by age-groups (B) in the DRC from 1990 to 2019.

**Additional file 6: Supplementary Figure 6. **Line plots showing age-standardised contribution of WASH factors to YLDs rate due to diarrhoeal diseases per 100,000 (A), and age-standardised contribution of WASH factors to deaths due to diarrhoeal diseases per 100,000 (B) in DRC from 1990 to 2019.

**Additional file 7: Supplementary File 7. **Distribution of YLDs (A) and deaths (B) per 100,000 people from diarrhoeal diseases by sex, age groups and WASH risk factors in DRC in 2019.

**Additional file 8: Supplementary File 8. **Plots showing contribution of children malnutrition risk factors to YLDs related with diarrhoeal diseases (A), and deaths (B) per 100000 population for under 5s in DRC from 1990 to 2019.

**Additional file 9: Supplementary File 9. **Distribution of YLDs (A) and deaths (B) per 100000 population from diarrhoeal diseases by sex and child nutrition factors in the DRC in 2019.

**Additional file 10: SupplementaryFigure 10. **Age-standardised YLDs (A) and age-standardised deaths (B) per 100000 by diarrhoeal diseases in DRC and neighbouring countries.

## Data Availability

The material used in this study and the data supporting these findings are available from the corresponding author on reasonable request. The dataset used/analysed in this current study is also available from the institute for Health Metrics and Evaluation (IHME) at the University of Washington at: http://ghdx.healthdata.org/gbd-results-tool.
