# Supplementary material for: The burden of diarrhoeal diseases in the Democratic Republic of Congo: a time-series analysis of the global burden of disease study estimates (1990–2019)
Source: BMC Public Health. 2022 May 25;22:1043. doi: 10.1186/s12889-022-13385-5 (PMC9131639; doi:10.1186/s12889-022-13385-5)

**SUPPLEMENTARY FILE 1**

**Appendix**

**Supplementary Figure 1.** Illustrating the mortality rate from diarrhoeal diseases in 2019 worldwide.^5^


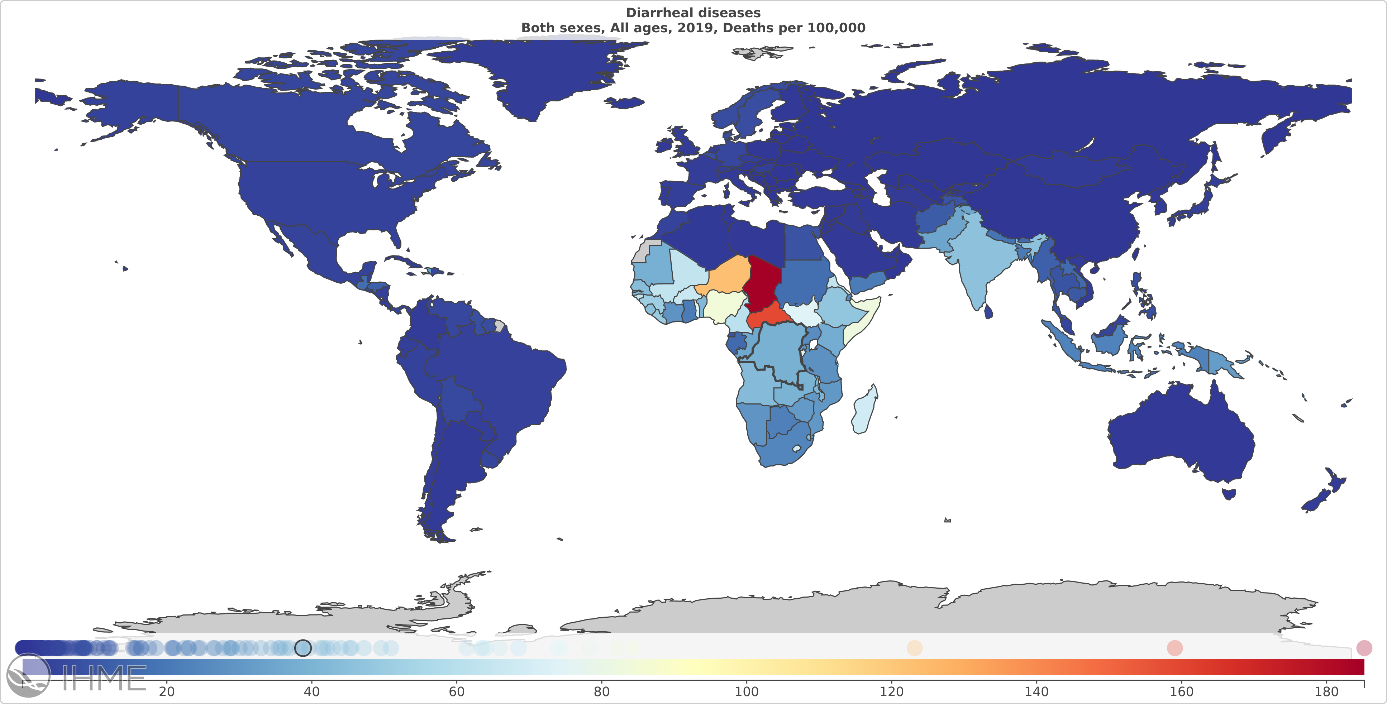

Supplement: Supplementary file 1 — Additional file 1: Supplementary Figure 1. Illustrating the mortality rate from diarrhoeal diseases in 2019 worldwide [5]. [file 12889_2022_13385_MOESM1_ESM.docx]
