# Supplementary material for: The burden of diarrhoeal diseases in the Democratic Republic of Congo: a time-series analysis of the global burden of disease study estimates (1990–2019)
Source: BMC Public Health. 2022 May 25;22:1043. doi: 10.1186/s12889-022-13385-5 (PMC9131639; doi:10.1186/s12889-022-13385-5)
Supplement: Supplementary file 2 — Additional file 2: Supplementary Figure 2. Line plots showing age-standardised prevalence of diarrhoeal diseases per 100,000 people, overall and by sex (A) and the overall prevalence by age-group (B), in DRC from 1990 to 2019. [file 12889_2022_13385_MOESM2_ESM.docx]

**SUPPLEMENTARY FILE 2**

**Supplementary Figure 2.** Line plots showing age-standardised prevalence of diarrhoeal diseases per 100,000 people, overall and by sex (A) and the overall prevalence by age-group (B), in DRC from 1990 to 2019.


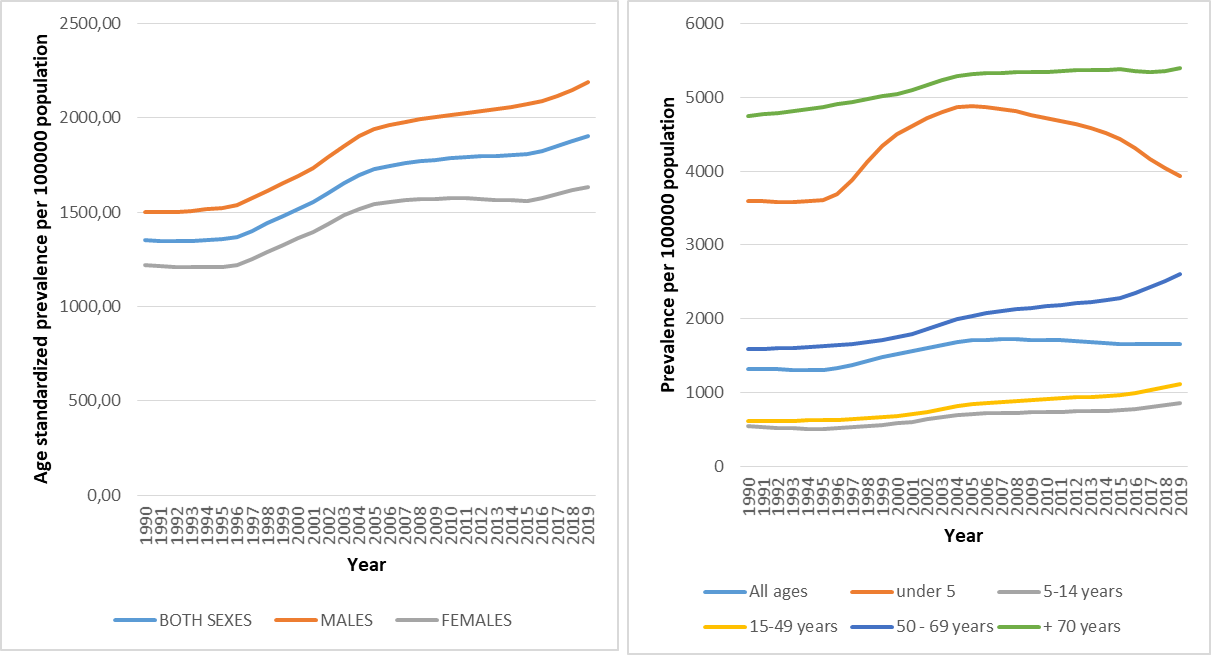


A

B
