# Supplementary material for: The burden of diarrhoeal diseases in the Democratic Republic of Congo: a time-series analysis of the global burden of disease study estimates (1990–2019)
Source: BMC Public Health. 2022 May 25;22:1043. doi: 10.1186/s12889-022-13385-5 (PMC9131639; doi:10.1186/s12889-022-13385-5)
Supplement: Supplementary file 3 — Additional file 3: Supplementary Figure 3. Arrow diagram showing the ranking of diseases by burden in DRC from 1990 to 2019 [27]. [file 12889_2022_13385_MOESM3_ESM.docx]

**SUPPLEMENTARY FILE 3**

**Appendix**

**Supplementary Figure 3.** Arrow diagram showing the ranking of diseases by burden in DRC from 1990 to 2019.^27^

**
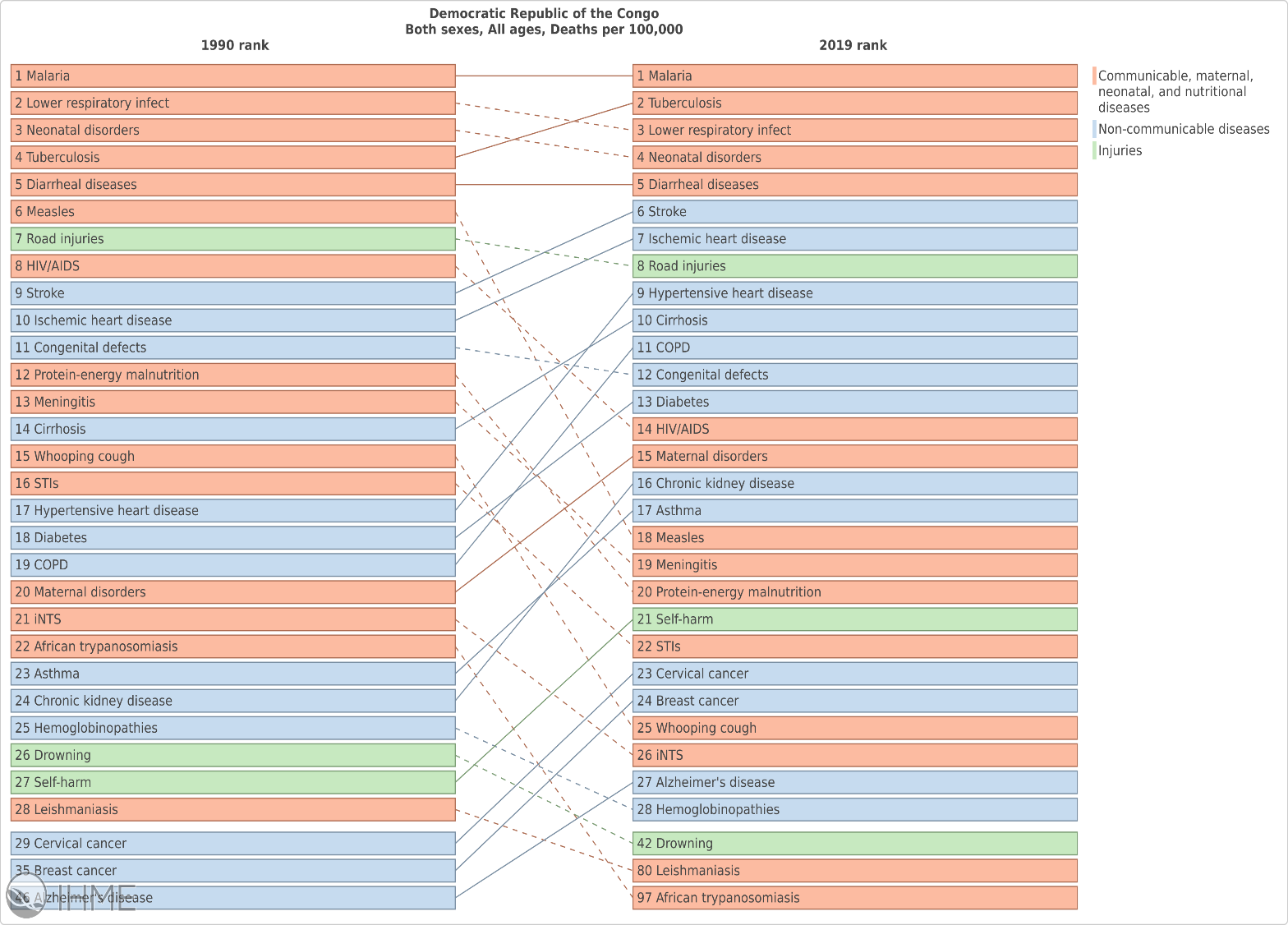
**
