# Supplementary material for: The burden of diarrhoeal diseases in the Democratic Republic of Congo: a time-series analysis of the global burden of disease study estimates (1990–2019)
Source: BMC Public Health. 2022 May 25;22:1043. doi: 10.1186/s12889-022-13385-5 (PMC9131639; doi:10.1186/s12889-022-13385-5)
Supplement: Supplementary file 5 — Additional file 5: Supplementary Figure 5. Line plots showing YLDs per 100,000 people, overall and by sex (A) and by age-groups (B) in the DRC from 1990 to 2019. [file 12889_2022_13385_MOESM5_ESM.docx]

**SUPPLEMENTARY FILE 5**

**Supplementary Figure 5.** Line plots showing YLDs per 100,000 people, overall and by sex (A) and by age-groups (B) in the DRC from 1990 to 2019.


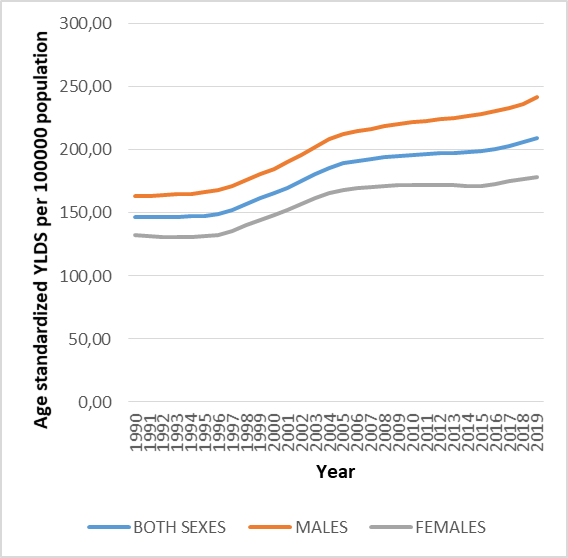

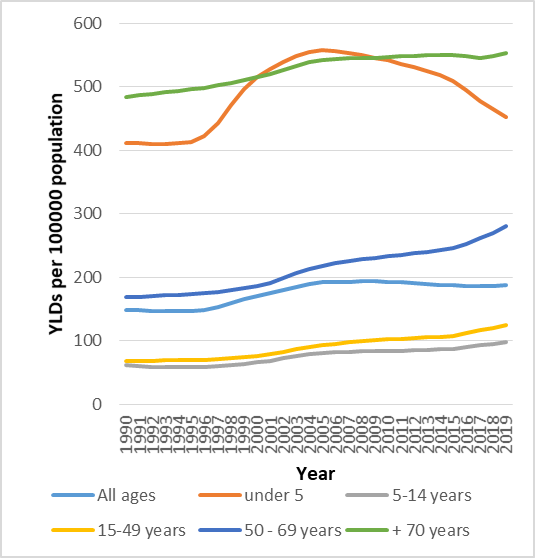


**B**

**A**
