# Supplementary material for: The burden of diarrhoeal diseases in the Democratic Republic of Congo: a time-series analysis of the global burden of disease study estimates (1990–2019)
Source: BMC Public Health. 2022 May 25;22:1043. doi: 10.1186/s12889-022-13385-5 (PMC9131639; doi:10.1186/s12889-022-13385-5)
Supplement: Supplementary file 6 — Additional file 6: Supplementary Figure 6. Line plots showing age-standardised contribution of WASH factors to YLDs rate due to diarrhoeal diseases per 100,000 (A), and age-standardised contribution of WASH factors to deaths due to diarrhoeal diseases per 100,000 (B) in DRC from 1990 to 2019. [file 12889_2022_13385_MOESM6_ESM.docx]

**SUPPLEMENTARY FILE 6**

Supplementary Figure 6. Line plots showing age-standardised contribution of WASH factors to YLDs rate due to diarrhoeal diseases per 100,000 (A), and age-standardised contribution of WASH factors to deaths due to diarrhoeal diseases per 100,000 (B) in DRC from 1990 to 2019.


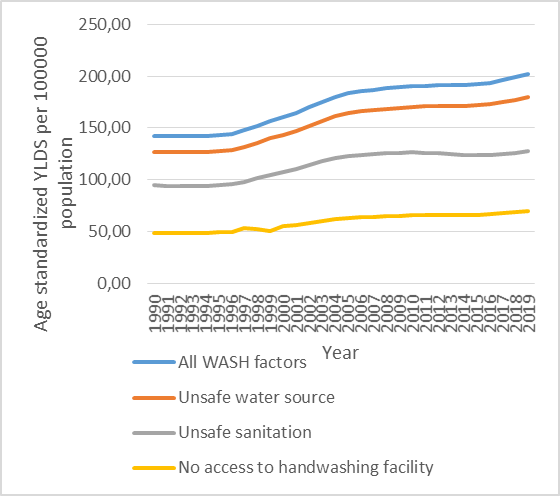

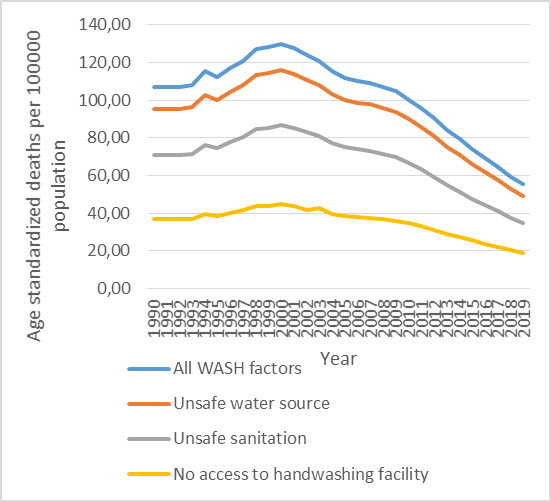


**B**

**A**
