# Supplementary material for: The burden of diarrhoeal diseases in the Democratic Republic of Congo: a time-series analysis of the global burden of disease study estimates (1990–2019)
Source: BMC Public Health. 2022 May 25;22:1043. doi: 10.1186/s12889-022-13385-5 (PMC9131639; doi:10.1186/s12889-022-13385-5)
Supplement: Supplementary file 8 — Additional file 8: Supplementary File 8. Plots showing contribution of children malnutrition risk factors to YLDs related with diarrhoeal diseases (A), and deaths (B) per 100000 population for under 5s in DRC from 1990 to 2019. [file 12889_2022_13385_MOESM8_ESM.docx]

**SUPPLEMENTARY FILE 8**

**Supplementary File 8.** Plots showing contribution of children malnutrition risk factors to YLDs related with diarrhoeal diseases (A), and deaths (B) per 100000 population for under 5s in DRC from 1990 to 2019


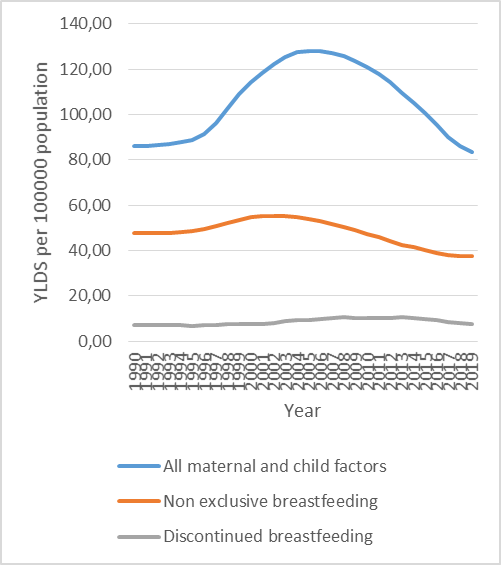

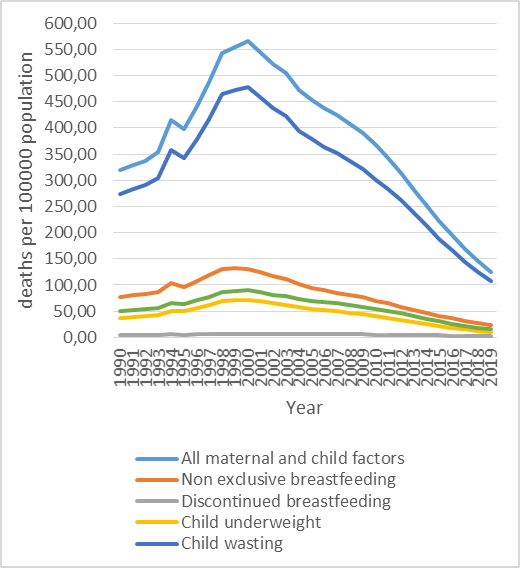


**B**

**A**
