# Supplementary material for: The burden of diarrhoeal diseases in the Democratic Republic of Congo: a time-series analysis of the global burden of disease study estimates (1990–2019)
Source: BMC Public Health. 2022 May 25;22:1043. doi: 10.1186/s12889-022-13385-5 (PMC9131639; doi:10.1186/s12889-022-13385-5)
Supplement: Supplementary file 9 — Additional file 9: Supplementary File 9. Distribution of YLDs (A) and deaths (B) per 100000 population from diarrhoeal diseases by sex and child nutrition factors in the DRC in 2019. [file 12889_2022_13385_MOESM9_ESM.docx]

**SUPPLEMENTARY FILE 9**

**Supplementary File 9.** Distribution of YLDs (A) and deaths (B) per 100000 population from diarrhoeal diseases by sex and child nutrition factors in the DRC in 2019.


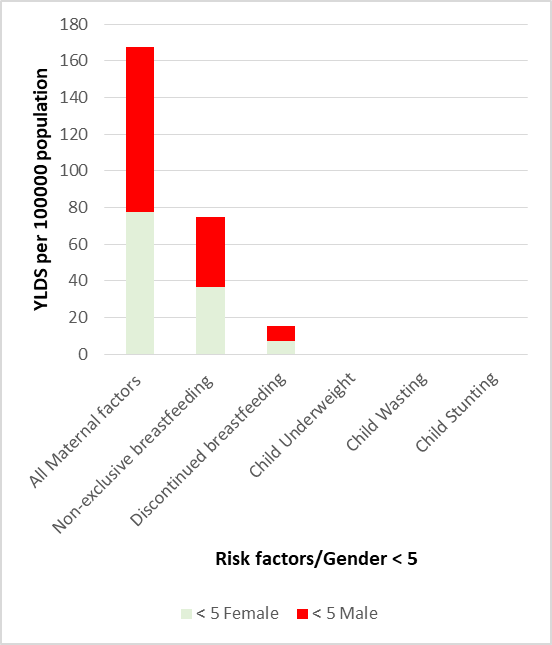

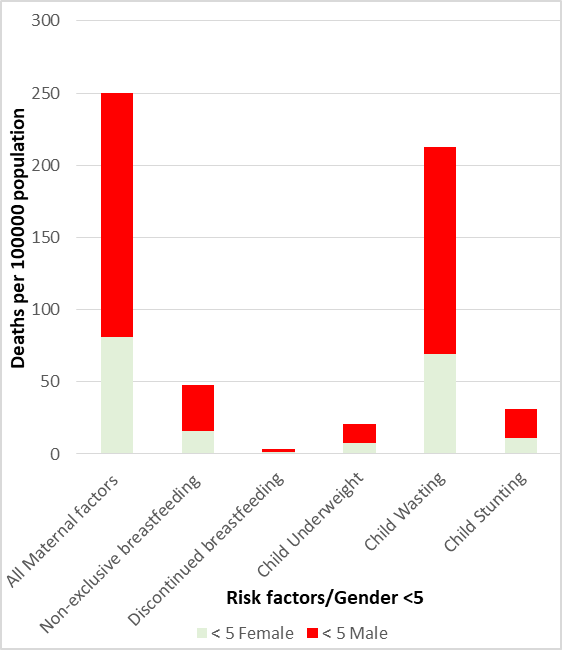


**B**

**A**
