# Supplementary material for: The burden of diarrhoeal diseases in the Democratic Republic of Congo: a time-series analysis of the global burden of disease study estimates (1990–2019)
Source: BMC Public Health. 2022 May 25;22:1043. doi: 10.1186/s12889-022-13385-5 (PMC9131639; doi:10.1186/s12889-022-13385-5)
Supplement: Supplementary file 10 — Additional file 10: SupplementaryFigure 10. Age-standardised YLDs (A) and age-standardised deaths (B) per 100000 by diarrhoeal diseases in DRC and neighbouring countries. [file 12889_2022_13385_MOESM10_ESM.docx]

**SUPPLEMENTARY FILE 10**

SupplementaryFigure 10. Age-standardised YLDs (A) and age-standardised deaths (B) per 100000 by diarrhoeal diseases in DRC and neighbouring countries.


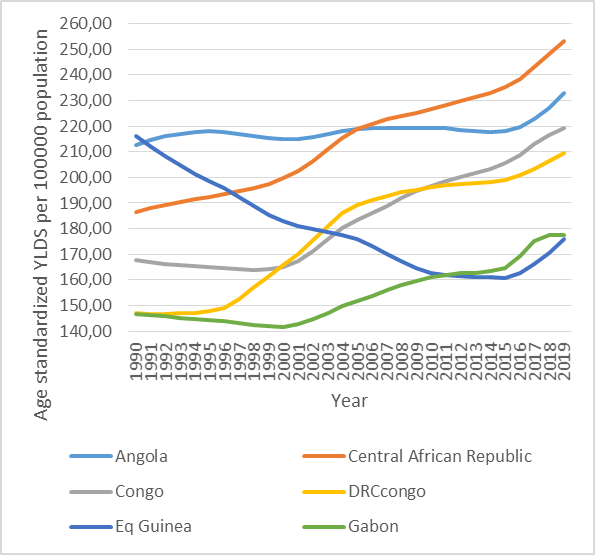

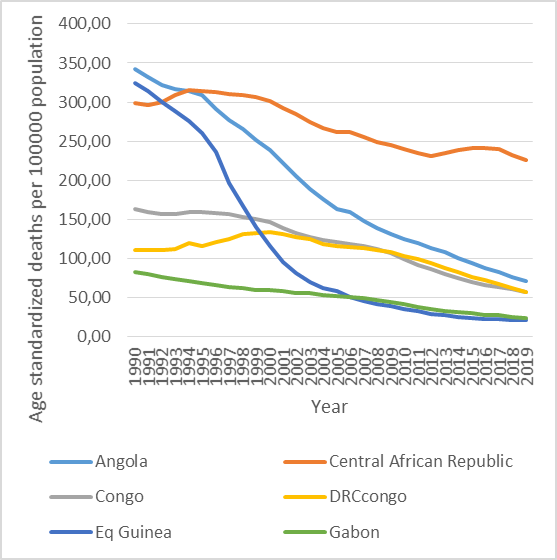


**B**

**A**
